# Impact of Timing of Beta-Lactam Therapeutic Drug Monitoring and Therapy Adjustment in Critically Ill Patients

**DOI:** 10.3390/antibiotics14050463

**Published:** 2025-05-01

**Authors:** Mohammad H. Alshaer, Nicole F. Maranchick, Kelly L. Maguigan, Bethany R. Shoulders, Mays J. Mousa, Melissa Murray, Jennifer Ashton, Kaitlin Alexander, Barbara A. Santevecchi, Kathryn DeSear, Veena Venugopalan, Kartikeya Cherabuddi, Charles A. Peloquin

**Affiliations:** 1 Department of Pharmacotherapy and Translational Research, College of Pharmacy, University of Florida, Gainesville, FL 32610, USA; n.maranchick@cop.ufl.edu (N.F.M.); brshoulders@cop.ufl.edu (B.R.S.); maysmousa30@gmail.com (M.J.M.); kaitlin.alexander@cop.ufl.edu (K.A.); bsantevecchi@ufl.edu (B.A.S.); vvenugopalan@cop.ufl.edu (V.V.); peloquin@ufl.edu (C.A.P.); 2 Emerging Pathogens Institute, University of Florida, Gainesville, FL 32610, USA; 3 Department of Pharmacy, UF Health Shands Hospital, Gainesville, FL 32608, USA; kelly.maguigan@gmail.com (K.L.M.); jmelir@shands.ufl.edu (M.M.); jennifer.ashton@ynhh.org (J.A.); deseak@shands.ufl.edu (K.D.); 4 Division of Infectious Diseases, College of Medicine, University of Florida, Gainesville, FL 32610, USA; kartikeya.cherabuddi@medicine.ufl.edu

**Keywords:** beta-lactam, early drug monitoring, clinical cure, mortality

## Abstract

**Purpose:** To assess the impact of beta-lactam therapeutic drug monitoring (TDM) timing and therapy adjustment on clinical cure and 30-day mortality. **Methods:** This was a prospective study of critically ill patients admitted to the University of Florida Health Shands Hospital intensive care unit (ICU) between 2021 and 2022, ≥18 years old, and requiring beta-lactam therapy for a suspected or confirmed infection. Beta-lactam concentrations were measured per standard of care, pharmacokinetic/dynamic (PK/PD) target attainment was calculated, and therapy was adjusted if needed. Multiple regression and time-to-event (TTE) analyses were performed. **Results:** A total of 297 infection episodes from 268 patients were included. The mean (SD) age was 56 years (17), weight was 82 kg (32), and 14% received renal replacement therapy. The most common infection source was the lung, and the most common beta-lactam was cefepime. The most common infusion duration was 30 min. The median (IQR) time to first TDM was 2.7 days (1.7–4.7). Fifty-seven percent of patients required therapy adjustment. Increases in beta-lactam dose, frequency, or infusion duration were associated with lower 30-day mortality compared to continuing the same regimen (aOR 0.30, *p* = 0.015). Delay in performing TDM was associated with lower probability of clinical cure (aOR 0.92, *p* = 0.0023). Patients who had the regimen increased had shorter hospital stay compared to those who had it decreased. Timing of beta-lactam TDM in ICU patients was a significant predictor of clinical cure, while adjusting beta-lactam therapy to achieve higher exposure was a significant predictor of 30-day mortality.

## 1. Introduction

Beta-lactams are one of the most commonly prescribed classes of antibiotics in hospitalized patients. A survey of antimicrobial use in United States hospitals showed 12.2% of patients received third- or fourth-generation cephalosporins, 9.8% received first-generation cephalosporin, 8.7% received penicillin combinations, and 3.7% received carbapenems in 2015 [1]. Preclinical studies have identified beta-lactams to be time dependent, meaning the time free beta-lactam concentrations remain above the minimum inhibitory concentration (*f*T_>MIC_) drives bacterial killing [2,3].

For critically ill patients with severe infections, early initiation of appropriate antibiotic therapy has shown to be an effective intervention [4,5,6,7]. Therapeutic drug monitoring (TDM) is a helpful tool to identify patients with suboptimal antibiotic exposure early in therapy. Previously reported beta-lactam pharmacokinetic/dynamic (PK/PD) targets in critically ill patients included 60% *f*T_>MIC_ and 100% *f*T_>1–5×MIC_ [2,8]. Such targets may not be achieved using doses reported in the package insert, as several factors can alter the pharmacokinetics of beta-lactams in critically ill patients, including severity of illness and organ dysfunction [8,9,10]. As a result, early optimization of beta-lactam therapy in critically ill patients is essential.

In this study, we aim to evaluate the impact of beta-lactam TDM timing and therapy adjustments on 30-day mortality, clinical cure, and hospital stay.

## 2. Results

During the study period, 297 episodes of infection from 268 ICU patients were included (Table 1). The mean (SD) age was 56 years (17), weight was 82 kg (32), and baseline serum creatinine was 1.26 mg/dL (1.14). More than half of patients (57%) were males, and 14% received RRT. The most common infection source was the lung (44%), and the most common isolated bacteria was *Pseudomonas aeruginosa* (n = 115). Breakpoint MICs were utilized in 79 (27%) of TDM instances. Table S1 lists all bacterial isolates and median MICs. Twenty-three percent received vancomycin concomitantly with beta-lactam therapy.

Table S2 summarizes the initial beta-lactam regimens, PK/PD targets, and therapy changes. The median (IQR) beta-lactam duration of therapy was 7 days (5–12). Most patients (91%) received their beta-lactam therapy as an intermittent infusion. The median time from starting beta-lactam therapy to the first TDM sample was 2.7 days (1.7–4.7). In terms of *f*T_>MIC_, the median target attainment was 100% for most beta-lactams; while the median target attainment was 100% in the case of *f*T_>4×MIC_ for ampicillin and meropenem only. This was reflected by the *f*C_min_/MIC ratio, which was high (median > 15) in ampicillin and meropenem; between 3 and 4 in cefazolin, cefepime, and piperacillin; and ≤2 in the case of aztreonam, ceftriaxone, and oxacillin. More than half of the patients (57%) had their beta-lactam regimen changed post TDM, split between an increase and decrease in the regimen. Thirty-two patients had a second beta-lactam TDM instance; 47% from the therapy increase group and 35% from the therapy decrease group. Patients achieving 100% *f*T_>MIC_ may still have had therapy increased if clinicians wanted a higher target, such as 100% *f*T_>4×MIC_. In the second TDM event, the median (IQR) *f*C_min_/MIC was 4.9 (1.8–14.9), *f*T_>MIC_ 100% (100–100), and *f*T_>4×MIC_ 23% (7–37).

Clinical cure was reported in 75% of infections, and 30-day mortality was 20%. The median ICU length of stay was 15 days (7–30), and the hospital length of stay was 22 days (13–41). Table 2 and Table 3 show the primary analyses for clinical cure and 30-day mortality, respectively. Figure S1 shows the initial target attainment by beta-lactam regimen change. For clinical cure, increases in days to TDM were significant predictors of worse therapy outcomes. For the 30-day mortality, SOFA score, RRT, age, intraabdominal infection source, and change in beta-lactam regimens were significant predictors of this outcome. Specifically, patients whose beta-lactam dose, frequency, and/or duration of infusion increased were less likely to die up to day 30 compared to those who continued the same regimen. We performed sensitivity analysis, including only patients requiring therapy change in the primary analysis, and days to TDM remained as a significant predictor of clinical cure (aOR [95% CI], 0.89 [0.82, 0.97]).

**Figure 1:**
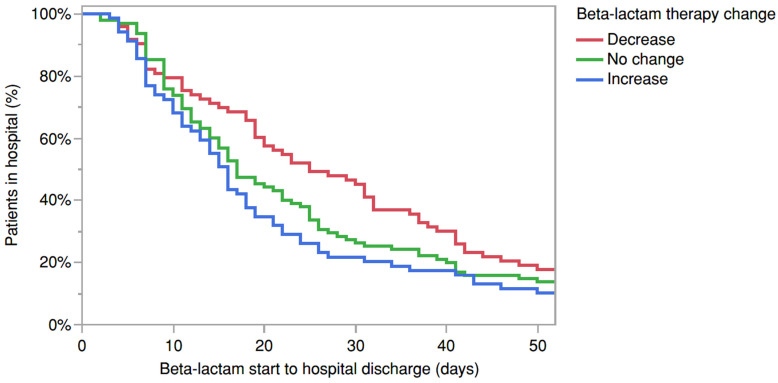
Kaplan–Meier curves showing the days from starting the beta-lactam to hospital discharge and the percentage of patients remaining in the hospital by the beta-lactam therapy change group (red = therapy decrease, green = no change in therapy, blue = therapy increase). Log-Rank *p* = 0.0446 (all-group comparison), Log-Rank *p* = 0.0137 (therapy decrease vs. increase comparison).

Figure 1 shows the Kaplan–Meier curve for the length of stay grouped by change in beta-lactam therapy. Patients who had therapy increased had shorter hospital stay compared to the other groups (Log-Rank *p* = 0.0446). The difference was most prominent between therapy increase and decrease groups (Log-Rank *p* = 0.0137).

## 3. Discussion

We reported clinical outcomes associated with an active beta-lactam TDM service in ICU patients. Patients had their beta-lactam plasma concentrations evaluated for therapy modification in a median of 2.7 days. Therapy adjustments and time to therapy change were evaluated as predictors of mortality and clinical cure. Lower mortality was observed in those who had their beta-lactam dose, frequency, and/or duration of infusion increased compared to those who continued the same regimen. This may reflect that higher target attainment might be associated with lower probability of 30-day mortality. In addition, patients who had therapy increased had shorter hospital stay compared to those who had therapy decreased in the TTE analysis, which could have benefits, including reducing the patient’s risk of hospital-acquired infection and costs. Unfortunately, target attainment was re-evaluated in a small number of patients, as this was based on the clinician’s discretion and not study or hospital protocol. The timing of measuring the beta-lactam concentration, including time to adjust therapy and the associated PK/PD target attainment after such therapy modification, was a significant predictor of clinical cure. This was confirmed by the sensitivity analysis performed, including only those who required therapy change.

Our results are consistent with a previously published cohort study of 206 ICU patients with measured beta-lactam concentration. In that study, delay in measuring beta-lactam concentration was associated with clinical failure, longer stay, and higher mortality at day 30. The study was majorly observational, with minimal therapy adjustment, as only 12 patients had their beta-lactam regimen changed; hence the beta-lactam PK/PD target attainment on the first measurement occasion was a predictor of clinical outcomes [11]. However, our study reported an active TDM service, where all patients had their therapy adjusted as needed based on the measured beta-lactam concentration. As a result, the PK/PD target attainment, which was on the first occasion only, was not a significant predictor of clinical outcomes, as therapy was adjusted in 57% of patients. On the other hand, optimizing beta-lactam regimen, which is expected to optimize PK/PD target attainment, was associated with lower probability of mortality. At the same time, the timing of TDM and therapy adjustment was important for clinical cure but not mortality.

Other studies reported early target attainment evaluation in ICU patients. Huttner and colleagues reported a prospective ICU study including 100 patients with creatinine clearance >60 mL/min. Beta-lactam concentrations were measured on days 1 to 3 and 5. Eighty-six percent of patients had subtherapeutic trough values, and undetectable beta-lactam trough concentrations were reported in 27% of patients [12]. Another prospective study evaluated the achievement of *f*T_>4×MIC_ after the first dose of beta-lactam in patients with severe sepsis and septic shock. The *P. aeruginosa* breakpoint was used for target attainment. In the 80 patients included in this study, *f*T_>4×MIC_ was 57% for meropenem, 45% for ceftazidime, 34% for cefepime, and 33% for piperacillin-tazobactam. The percentage of patients who achieved the desired PK/PD target was, for meropenem (75%), ceftazidime (28%), cefepime (16%), and piperacillin-tazobactam (44%) [13].

Many previous studies investigated the impact of beta-lactam PK/PD target attainment in critically ill patients on therapy outcomes. A prospective, multicenter study included 361 ICU patients who received one of eight beta-lactam therapies. Piperacillin and meropenem were the top two. Patients had mid-dose and trough blood samples drawn to calculate the 50% and 100% *f*T_>MIC_, respectively. Investigators found that both 50% and 100% *f*T_>MIC_ were associated with a positive clinical outcome, while controlling for SOFA and Acute Physiology and Chronic Health Evaluation II scores [10]. Another multicenter trial randomized 249 septic patients receiving piperacillin-tazobactam continuous infusion to either a TDM-guided or fixed-dose arm and evaluated the mean daily total SOFA score as the primary outcome. The investigators did not find a difference in terms of mean SOFA scores, 28-day mortality, and clinical and microbiologic cure. However, the piperacillin exposure was similar between the arms, which may explain the similarity in the clinical outcomes [14]. Alshaer et al. evaluated the clinical outcomes in ICU patients who received cefepime, meropenem, or piperacillin-tazobactam and had pneumonia. The investigators used machine learning to rank the predictors of clinical outcomes. A total of 735 patients and 840 pneumonia episodes were included in this study. Both multiple regression and machine learning showed that PK/PD target attainment was a significant predictor of clinical cure, and the machine learning showed that *f*T_>4×MIC_, especially in the first 24 h of therapy, was the top predictor of clinical cure in these patients [15]. Similarly, another study included 204 patients with bloodstream infection who received cefepime, meropenem, or piperacillin-tazobactam. In the multiple regression analyses, *f*T_>4×MIC_ from time 0 to 24 h and from 0 to 7 days was a significant predictor of microbiologic eradication, and patients who achieved 100% *f*T_>4×MIC_ had significantly shorter time to negative blood culture in the TTE analysis [16]. As a continuation of the previous published work, we evaluated the timing of therapy change, which proved to be an important predictor of clinical cure in our study. This means that not only do clinicians need to optimize beta-lactam therapy, they also need to do this early in therapy.

Our study had a number of limitations. First, it included multiple beta-lactams, covering a broad spectrum of bacteria. However, the majority of the patients received cefepime or meropenem, which may make the study more generalizable to Gram-negative infections. Second, only total beta-lactam concentrations were measured, and unbound fraction was estimated using published values, which may not be reflective of ICU patients’ values. In addition, fT_>MIC_ uses concentrations from the blood, not directly at the site of infection. Finally, remeasuring beta-lactam concentration to evaluate target attainment after therapy modification was not part of the protocol and was left to the clinician’s discretion. We suggest that future studies evaluating the timing of beta-lactam TDM intervention address these limitations to generate more robust data.

In ICU patients, the timing of beta-lactam TDM was a significant predictor of clinical cure, while adjusting beta-lactam therapy to achieve higher exposure was a significant predictor of 30-day mortality and shorter hospital stay. As a result, given the turnaround time of the drug quantification, it is recommended to measure beta-lactam concentration as early as possible, preferably in the first few hours of therapy, to allow for timely reporting of the concentration so therapy can be optimized early on.

## 4. Methods

This was a prospective, observational study that aimed to identify the clinical outcomes in patients receiving beta-lactam therapy with TDM admitted to University of Florida (UF) Health Shands intensive care unit (ICU) between June 2021 and December 2022. Patients were included if they were ≥18 years old, had suspected or confirmed bacterial infection, received beta-lactam therapy including ampicillin (EUGIA, East Windsor, NJ, USA), aztreonam (ER SQUIBB, New Brunswick, NJ, USA), cefazolin (APOTEX, Weston, FL, USA), cefepime (APOTEX, Weston, FL, USA), ceftriaxone (PFIZER, New York, NY, USA), meropenem (WG CRITICAL CARE, Paramus, NJ, USA), oxacillin (FRESENIUS KABI, Lake Zurich, IL, USA), or piperacillin (FRESENIUS KABI, Lake Zurich, IL, USA), and had beta-lactam plasma concentration(s) measured. Exclusion criteria included pregnancy, incarcerated patients, and patients with resistant infections.

Data collected included patient age, sex, renal replacement therapy (RRT), Sequential Organ Failure Assessment (SOFA) scores, cultures and susceptibilities, site of cultures, infection sources, beta-lactam regimens and plasma concentrations, therapy outcomes, hospital and ICU admission/discharge dates, and death date. SOFA and RRT represent variables that demonstrate organ impairment or dysfunction, which are significant contributors to mortality. Furthermore, these variables contribute to changes in pharmacokinetics that impact the pharmacodynamics of antimicrobials (e.g., renal clearance), which may be difficult to predict, requiring a personalized approach to antimicrobial dosing.

Patients were started on beta-lactam therapy using empirical doses based on their age, weight, and renal function. Empiric dosing for patients on RRT was based on standardized hospital protocols. Patients had their beta-lactam concentrations measured and repeated per clinician discretion [17]. Pharmacists ordered peak (1 h after the end of the infusion) and trough (before the start of next dose) samples for intermittent and extended infusions. For continuous infusions, one to two random samples were collected. Total beta-lactam plasma concentrations were quantified at the Infectious Disease Pharmacokinetics Lab at UF using validated liquid chromatography with tandem mass spectrometry assays. Beta-lactam quantification was performed Monday through Friday for ampicillin, cefazolin, cefepime, piperacillin, meropenem, and oxacillin and as needed for amoxicillin, aztreonam, ceftriaxone, imipenem, and nafcillin. The protein binding fraction was assumed to be 20% (ampicillin and cefepime), 56% (aztreonam), 80% (cefazolin), 90% (ceftriaxone), 2% (meropenem), 95% (oxacillin), and 30% (piperacillin), based on previously published values [18,19,20,21,22]. PK/PD target attainment was calculated using first-order pharmacokinetic equations, starting with calculating the rate of elimination. Then, the amount of time the free beta-lactam concentration was above the MIC and the free minimum concentration-to-MIC (*f*C_min_/MIC) ratios were calculated. Using the PK/PD data generated, beta-lactam therapy was adjusted within 24 h of receiving results based upon clinician discretion to target 100% *f*T_>1–4×MIC_. Patients with 100% *f*T_>MIC_ may have had dose increases to achieve a higher target of *f*T_>4×MIC_, depending upon infection severity, infection type, and suspected organism.

The MICs for the bacteria were determined in the onsite UF Health Shands microbiology laboratory. Bacteria were identified by standard microbiologic methods using Vitek^®^ II (bioMérieux, Inc., Durham, NC, USA). For polymicrobial infections, the highest reported MIC was used for PK/PD calculations. When using cefazolin to treat methicillin-susceptible *Staphylococcus aureus*, a breakpoint of 2 mg/L was considered as the target. If no bacteria were isolated, the Clinical and Laboratory Standards Institute breakpoint for the suspected causative pathogen was used.

Since all patients had TDM performed and therapy adjusted accordingly, the primary outcome of this study was the impact of beta-lactam TDM timing and therapy adjustment on clinical cure and 30-day mortality. The secondary outcome was hospital length of stay. Clinical cure was defined as the resolution of infection-related symptoms at the end of therapy (including normalization of body temperature, WBC count, and discontinuation of mechanical ventilation and/or vasopressors) without change or addition of antibiotic therapy and non-initiation of a new antibiotic within 48 h of stopping the original agent. De-escalation to a narrower spectrum antibiotic was allowed and not considered clinical failure. Beta-lactam therapy increase was defined as increase in daily dose, frequency, and/or infusion duration, while therapy decrease was defined as decrease in daily dose, frequency, and/or infusion duration.

### Statistical Analysis

Continuous data were summarized as mean and standard deviation (SD) or median and interquartile range (IQR), and categorical data as counts and percentages. Multiple logistic regression was performed, and adjusted odds ratios (aORs) along with 95% confidence intervals (CIs) were reported. Predictors previously known to impact clinical outcomes were included in the models, including SOFA scores, age, infection source, and RRT [15,16]. The predictors tested in these analyses were time from starting therapy to measuring concentration (continuous variable) and change in beta-lactam therapy after performing TDM (ordinal variable coded as 1 for increase in regimen; 0 for no change in therapy; and −1 for decrease in regimen). Time-to-event (TTE) analysis was performed, and Kaplan–Meier curves were reported for time to discharge while excluding patients with 30-day mortality. Patients with a hospital stay longer than 50 days were censored in the TTE analysis. A *p*-value < 0.05 was considered statistically significant. JMP^®^ Pro Version 16.1 (SAS Institute Inc., Cary, NC, USA, 1989–2023) was used for statistical analysis.

## Figures and Tables

**Table 1:** Demographic and clinical characteristics of patients, n = 268. Mean (SD) or n (%).

Characteristic	Total (n = 268)	Increase (n = 71)	Decrease (n = 79)	No Change (n = 118)	*p*-Value
Age, years	56 (17)	55 (18)	58 (15)	56 (18)	0.32
Weight, kg	82 (32)	84 (35)	86 (38)	77 (24)	0.07
Male	152 (57)	48 (68)	36 (46)	68 (58)	0.10
Baseline serum creatinine, mg/dL	1.26 (1.14)	0.95 (0.82)	1.45 (0.93)	1.27 (1.31)	0.05
Baseline SOFA score	6 (4)	5 (3)	6 (4)	6 (4)	0.13
ICU length of stay, days	15 (7–30)	24 (38)	24 (23)	23 (31)	0.78
Hospital length of stay, days	22 (13–41)	29 (39)	32 (31)	31 (34)	0.61
Renal replacement therapy, yes	38 (14)	7 (10)	8 (10)	23 (19)	0.08
Most common infection source	N = 297	N = 75	N = 93	N = 129	
Lung	131 (44)	28 (37)	38 (41)	65 (50)	0.15
Bacteremia	51 (17)	14 (19)	18 (19)	19 (15)	0.62
Skin/soft tissue	26 (9)	10 (13)	10 (11)	6 (5)	0.08
Intra-abdominal	20 (7)	1 (1)	9 (10)	10 (8)	0.08
Urinary tract	18 (6)	3 (4)	5 (5)	10 (8)	0.53
Bone/joint	14 (5)	8 (11)	3 (3)	3 (2)	0.02
Endocarditis	12 (4)	3 (4)	4 (4)	5 (4)	0.99
Concomitant antimicrobials					
Aminoglycoside	9 (3)	1 (1)	3 (3)	5 (4)	0.31
Daptomycin	6 (2)	0 (0)	2 (2)	4 (3)	0.37
Fluoroquinolone	2 (<1)	0 (0)	0 (0)	2 (20)	0.99
Linezolid	24 (8)	1 (1)	10 (11)	13 (10)	0.05
TMP/SMZ	9 (3)	0 (0)	3 (3)	6 (5)	0.88
Vancomycin	68 (23)	19 (25)	18 (19)	31 (24)	0.57
Most common isolated bacteria ^†^					
*Pseudomonas aeruginosa*	115 (2)	37 (4)	34 (1)	44 (3)	0.84
*Escherichia coli*	46 (1)	12 (2)	19 (0.25)	15 (1)	0.14
*Klebsiella pneumoniae*	39 (1)	4 (0.25)	17 (1)	18 (1)	0.04
*Staphylococcus aureus*	30 (2)	21 (0.5)	2 (1.25)	7 (2)	<0.0001
*Enterobacter cloacae*	26 (1)	3 (1)	6 (1)	17 (1)	0.03
*Proteus mirabilis*	16 (1)	3 (1)	10 (1)	3 (1)	0.03
*Serratia marcescens*	14 (1)	2 (1.5)	9 (1)	3 (1)	0.04
Empiric Breakpoints used, n	79 (26)	22	20	37	0.17

SOFA, sequential organ failure assessment; ICU, intensive care unit. ^†^ Data presented as isolate count (median minimum inhibitory concentration).

**Table 2:** Univariate and multiple regression analysis for clinical cure.

Predictors	Univariate Analysis—Clinical Cure, ORs (95% CI)	Multiple Regression—Clinical Cure, aORs (95% CI)
SOFA score	0.94 (0.88, 1.003)	-
RRT, yes	0.57 (0.30, 1.11)	-
Age	0.99 (0.97, 1.002)	-
Days to TDM	0.92 (0.88–0.98) *	0.92 (0.88, 0.98) *
Baseline SCr, mg/dL	0.91 (0.73–1.13)	-
Regimen change		
Increase vs. no change	-	1.17 (0.60, 2.30)
Decrease vs. no change	-	1.25 (0.67, 2.33)
Increase vs. decrease	-	0.94 (0.45, 1.95)

* Statistically significant result. RRT, renal replacement therapy; SOFA, sequential organ failure assessment; TDM, therapeutic drug monitoring; SCr, serum creatinine.

**Table 3:** Univariate and multiple regression analysis for 30-day mortality.

Predictors	Univariate Analysis, ORs (95% CI)	Multivariate Analysis, aORs (95% CI)
**SOFA score**	1.17 (1.09, 1.27) *	1.14 (1.04, 1.25)*
**RRT,** yes	3.59 (1.85, 6.96) *	2.07 (0.91, 4.67)
**Age**	1.04 (1.02, 1.06) *	1.05 (1.02–1.07) *
**Days to TDM**	0.93 (0.86, 1.02)	-
**Baseline SCr, mg/dL**	1.17 (0.94, 1.46)	-
**Infection source, intra-abdominal**	4.54 (1.79, 11.49) *	4.82 (1.53, 15.21) *
**Regimen change**		
Increase vs. no change	-	0.36 (0.13, 0.97) *
Decrease vs. no change	-	0.67 (0.33, 1.35)
Increase vs. decrease	-	0.45 (0.16, 1.24)

* Statistically significant result. RRT, renal replacement therapy; SOFA, sequential organ failure assessment; TDM, therapeutic drug monitoring; SCr, serum creatinine.

## Data Availability

The raw data supporting the conclusions of this article will be made available by the authors on request.

## Supplementary Materials

The following supporting information can be downloaded at: https://www.mdpi.com/article/10.3390/antibiotics14050463/s1, Table S1. Bacterial isolates and median MICs, n = 433; Table S2. Initial beta-lactam regimens, target attainment, and therapy changes; Figure S1. Pharmacokinetic/pharmacodynamic target attainment, including fT_>MIC_, fT_>4×MIC_, and fC_min_/MIC, on the first TDM occasion, grouped by change in beta-lactam therapy.
