# Supplementary material for: Impact of Timing of Beta-Lactam Therapeutic Drug Monitoring and Therapy Adjustment in Critically Ill Patients
Source: Antibiotics (Basel). 2025 May 1;14(5):463. doi: 10.3390/antibiotics14050463 (PMC12108447; doi:10.3390/antibiotics14050463)

Supplementary Material

Table S1: Bacterial isolates and median MICs, n=433

**Bacteria**	**Count**	**Median MIC, mcg/mL**
Pseudomonas aeruginosa	115	2
No growth / Empiric	79	8
Escherichia coli	46	1
Klebsiella pneumoniae	39	1
Staphylococcus aureus	30	2
Enterobacter cloacae	26	1
Proteus mirabilis	16	1
Acinetobacter baumannii	14	2
Serratia marcescens	14	1
Enterococcus faecalis	9	2
Klebsiella aerogenes	6	0.625
Citrobacter freundii	4	1
Achromobacter xylosoxidans	3	16
Klebsiella oxytoca	3	1
Pseudomonas putida	3	2
Bacteroides fragilis	2	1
Chryseomonas luteola	2	0.25
Clostridium perfringens	2	2
Kluyvera intermedia	2	8
Microaerophilic Streptococcus	2	1
Providencia stuartii	2	0.625
Acinetobacter iwoffii	1	1
Alcaligenes faecalis	1	0.25

Burkholderia cepacia	1	8
Citrobacter koseri	1	1
Pantoea agglomerans	1	1
Proteus penneri	1	1
Proteus species	1	1
Pseudomonas fluorescens	1	6
Raoultella planticola	1	1
Raoultella species	1	0.25
Staphylococcus epidermidis	1	2
Staphylococcus simulans	1	2
Staphylococcus species, coagulase negative	1	2

Table S2: Initial beta-lactam regimens, target attainment, and therapy changes†

**Parameter**	**All (n=297)**	**Ampicillin (n=6)**	**Aztreonam (n=2)**	**Cefazolin (n=16)**	**Cefepime (n=190)**	**Ceftriaxone (n=1)**	**Meropenem (n=55)**	**Oxacillin (n=6)**	**Piperacillin‡ (n=21)**
Daily dose, g	Variable	10 (9-12)	7 (2-12)	6 (3.25-6)	4 (4-6)	2	3 (3-4)	12 (10-12)	12 (12-16)
Infusion									
Intermittent	270 (91)	1 (17)	1 (50)	16 (100)	181 (95)	1 (100)	51 (93)	2 (33)	17 (81)
Extended	3 (1)	0	0	0	2 (1)	0	1 (2)	0	0
Continuous	24 (8)	5 (83)	1 (50)	0	7 (4)	0	3 (5)	4 (67)	4 (19)

Time to TDM[§], days	2.7 (1.7-4.7)	4.3 (2.6-9.4)	4.1 (2.2-6)	2.4 (1.7-4.8)	2.8 (1.7-4.7)	0.8	2.1 (1.6-3.6)	3.6 (3.2-4.7)	2.5 (1.7-5.8)
TDM samples[¶]									
One	40 (13)	1 (17)	0	2 (13)	20 (11)	0	9 (16)	3 (50)	5 (24)
Two	257 (87)	5 (83)	2 (100)	14 (87)	170 (89)	1 (100)	46 (84)	3 (50)	16 (76)
Target MIC, mg/L	2 (1-8)	2 (2-5)	10 (8-12)	2 (2-2)	4 (1-8)	4	0.5 (0.25-2)	0.5 (0.4-0.88)	8 (4-16)
$fT_{>MIC}$, %	100 (100-100)	100 (100-100)	100 (100-100)	100 (100-100)	100 (100-100)	34	100 (100-100)	100 (71-100)	100 (100-100)
$fT_{>4\times MIC}$, %	100 (50-100)	100 (100-100)	30 (0-59)	76 (44-97)	96 (46-100)	0	100 (100-100)	16 (0-51)	98 (34-100)
fC_{min}/MIC	4.4 (1.9-12)	15.6 (7.1-48.5)	2.6 (2.1-3.1)	3.1 (1.7-7.2)	3.8 (1.7-8.5)	0.6	15.4 (6.8-48.8)	1.4 (0.4-6.4)	4 (1.8-9.9)
Regimen change[#]									
Increase	75 (25)	0	0	9 (56)	51 (27)	1 (100)	5 (9)	5 (83)	4 (19)
No change	129 (43)	3 (50)	1 (50)	5 (31)	89 (47)	0	19 (35)	1 (17)	11 (52)
Decrease	93 (32)	3 (50)	1 (50)	2 (13)	50 (26)	0	31 (56)	0	6 (29)

†Reported as median (IQR) or n (%) unless specified otherwise

‡ Administered in combination with tazobactam

§ Time in days from starting beta-lactam therapy to drawing first sample for TDM

¶ Number of plasma samples drawn per TDM occasion

# Therapy increase is increase in total daily dose, frequency, and/or infusion time whereas therapy decrease is decrease in total daily dose and/or frequency.

Fig. S1 Pharmacokinetic/pharmacodynamic target attainment, including $fT_{>MIC}$, $fT_{>4×MIC}$, and fC_{min}/MIC, on the first TDM occasion grouped by change in beta-lactam therapy. The fC_{min}/MIC Y-axis is log-scaled. *fCmin/MIC* free minimum concentration to MIC ratio, *fT>MIC* time the free concentration remained above the MIC, *fT>4×MIC* time the free concentration remained above four multiples of the MIC, *Med* median, *MIC* minimum inhibitory concentration, *Max* maximum, *Min* minimum, *Q1* first quartile, *Q3* third quartile,

TDM therapeutic drug monitoring.

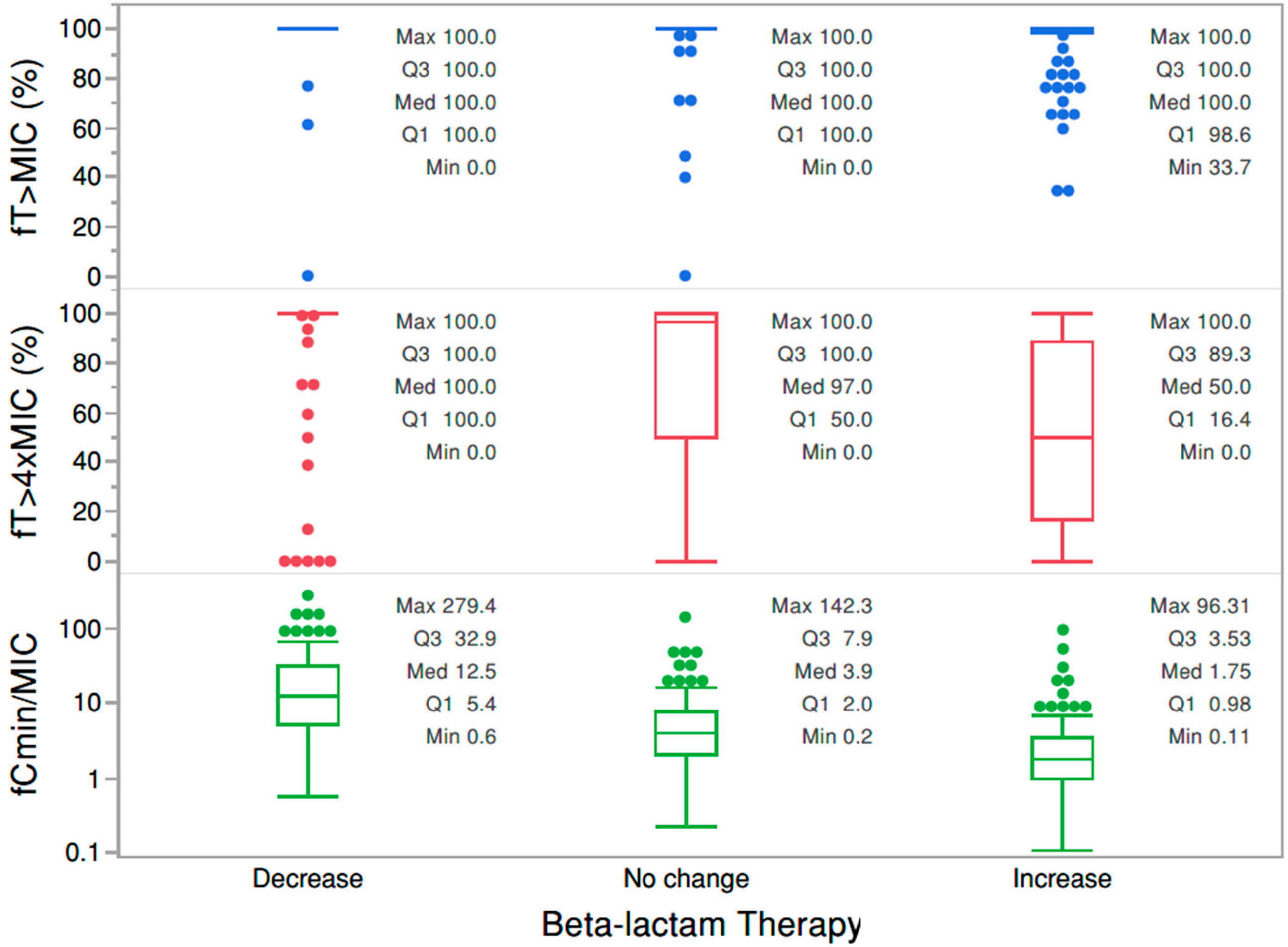

Supplement: Supplementary file 1 [file antibiotics-14-00463-s001.zip › antibiotics-3599886-supplementary.pdf]
